# Genome-Wide Association Study of Plant and Ear Height in Maize (*Zea mays* L.) and Identification of Candidate Genes

**DOI:** 10.3390/plants15091383

**Published:** 2026-04-30

**Authors:** Jiahao Wang, Yujia Zhang, Xinping Guo, Hexuan Liu, Liangliang Bao, Yuyang Zhou, Chunxiang Li, Hong Di

**Affiliations:** Key Laboratory of Germplasm Enhancement, Physiology and Ecology of Food Crops in Cold Region, Engineering Technology Research Center of Maize Germplasm Resources Innovation on Cold Land of Heilongjiang Province, Northeast Agricultural University, Harbin 150030, China; 17332328082@163.com (J.W.); zhangyujia7177@163.com (Y.Z.); 17662415546@163.com (X.G.); liuhexuan11121@163.com (H.L.); 15794892115@163.com (L.B.); zyyneau@163.com (Y.Z.)

**Keywords:** *Zea mays* L., plant height, ear height, GWAS, candidate genes, bioinformatics analysis

## Abstract

Maize is one of the most widely cultivated crops worldwide and is extensively used for animal feed and industrial applications. Plant height (PH) and ear height (EH) are critical determinants of lodging resistance and tolerance to high planting density, and coordinated regulation of these traits is essential for yield improvement. In this study, 479 maize inbred lines from Northeast and North China were genotyped using 7861 single-nucleotide polymorphism (SNP) markers to perform a genome-wide association study (GWAS). After controlling for population structure and relatedness, the mixed linear model (MLM) identified 20 loci significantly associated with PH on chromosomes 2, 4, 5, 6, 7, and 8, and 8 loci associated with EH on chromosomes 2, 3, 4, and 7. A total of 23 candidate genes were identified, including PLATZ8, pectin methylesterase 36, and leucine-rich repeat extensin 14. Gene Ontology (GO) enrichment analysis revealed significant enrichment in biological and molecular functions such as DNA binding, pectinesterase activity, zinc ion binding, ATP binding, and uniporter activity. Bioinformatic characterization of the two most likely candidate genes, *Zm00001d002726* and *Zm00001d015394*, showed that both possess a typical compact four-exon structure. Functional prediction indicated that *Zm00001d002726* encodes a pectinesterase/pectinase, potentially regulating cell elongation through pectin degradation and remodeling of the cell wall. Pectinesterase activity may influence PH and EH by mediating pectin demethylation within the cell wall. In contrast, *Zm00001d015394* encodes a PLATZ family transcription factor that may regulate downstream gene expression through DNA-binding activity. These findings provide insight into the genetic architecture and potential molecular mechanisms underlying PH and EH in maize and offer a foundation for future breeding efforts.

## 1. Introduction

Maize is one of the most important food and cash crops worldwide and serves as a primary raw material for food, feed, and industrial products [1]. In China, maize is the predominant grain crop, and increasing its yield is essential for ensuring national food security. Under the combined pressures of climate change and the continuous reduction of arable land, breeding new varieties with enhanced tolerance to high planting density and improved lodging resistance has become a major direction in the development of the maize industry [2]. The Green Revolution of the twentieth century substantially increased grain yield by reducing plant height (PH) and ear height (EH) to improve lodging resistance [3], in combination with advances in fertilization and irrigation technologies [4]. This integrated strategy represented a major turning point in modern agriculture. Central to this transformation was the use of dwarfing genes to modify crop architecture, thereby redirecting photosynthates from excessive vegetative growth toward grain accumulation [5]. A representative example is the elite maize inbred line D5003, from which a dwarfing gene was isolated and subsequently introgressed into multiple Chinese maize inbred lines, leading to the development of several dwarf varieties [6].

The concept of an “ideal plant architecture” was first proposed by Donald in 1968, emphasizing that optimization of morphological traits—such as leaf angle and PH distribution—can enhance population-level photosynthetic efficiency and stress tolerance [7]. As a structural optimization model, this concept aims to achieve high yield, stress resilience, and suitability for mechanized harvesting. Its central principle is the coordinated regulation of key traits, including PH and EH, to balance canopy structure and mechanical stability [8].

PH and EH are pivotal agronomic traits that determine maize lodging resistance, adaptability to high planting density [9], and harvesting efficiency [10]. Lodging significantly reduces maize yield and quality while also making mechanical harvesting more difficult [11]. Stalk lodging causes annual yield losses of 5–25% in the United States, according to estimates [12]. Lodging resistance is closely related to PH: taller plants have a higher center of gravity, which increases the bending moment on the stem, making them more susceptible to stalk breakage or root lodging [13]. Under high-density planting conditions, mutual shading among plants triggers shade avoidance syndrome, leading to increased PH and EH, reduced resistance to biotic and abiotic stress, decreased lodging resistance, and ultimately yield loss. With the widespread adoption of high-density planting to enhance yield per unit area, breeding maize varieties with optimal PH and ear height has become a critical breeding objective. Studies have shown that appropriately reducing PH and EH can enhance stem strength, improve lodging resistance, and facilitate mechanical harvesting [14].

These traits function in coordination rather than independently and must maintain an appropriate proportional relationship. Maintaining PH and EH within optimal ranges while preserving their coordination promotes redistribution of photosynthetic products, and strengthens both physiological and mechanical support for stable high yields. Consequently, a balanced combination of these traits contributes to ideal plant architecture, improving yield, reducing lodging risk, and supporting large-scale mechanized production for high-yield and high-quality outcomes.

Recent studies have demonstrated the effectiveness of the 60K SNP array for association analysis of maize plant architectural traits. In a population of 300 F1 individuals genotyped with this array, 17,652 high-quality SNP markers were identified, and subsequent GWAS detected nine loci significantly associated with PH, leading to the identification of two candidate genes [15,16]. Using F_1_ populations in genome-wide association analyses, a previous study further identified 108 candidate genes potentially involved in maize structural development, offering valuable resources for marker-assisted breeding [17]. PH, EH, and leaf number were evaluated across three environments using a natural population that included 226 inbred lines and 150 families derived from the cross T32 × Qi319. Through GWAS using a mixed linear model and linkage analysis using an inclusive composite interval mapping model, a total of 120 quantitative trait nucleotides (QTNs) and 32 QTLs were identified, of which 40 QTNs were associated with PH and 41 QTNs were associated with EH, highlighting the advantages of integrating multiple analytical approaches [18]. A total of 147 SNPs and 22 QTLs significantly associated with PH were identified using a multi-parent population consisting of 917 recombinant inbred lines (RILs) combined with whole-genome resequencing data, further enriching the genetic network underlying maize PH [19]. By analyzing the phenotypic plasticity of PH and EH across five contrasting environments, another study identified 77 loci exhibiting environmental interactions, highlighting the importance of multi-environment GWAS [20].

In addition to these findings, several key regulatory genes and modules associated with PH have been characterized. The *ZmCPK39–ZmKnox2* regulatory module coordinates hormone signaling with photosynthetic pathways and directly influences PH, providing potential targets for improving tolerance to high-density planting and lodging resistance [21]. The maize gene *ZmGF14-3* has also been identified as a regulator of PH; its overexpression in *Arabidopsis* results in dwarf phenotypes accompanied by reduced gibberellin and auxin levels and decreased expression of related genes, indicating that *ZmGF14-3* modulates PH through hormone signaling pathways [22]. *ZmCOLD1*, a GPCR-like protein, regulates plant growth via interaction with the Gα subunit *ZmCT2* through its hydrophilic loop and participates in abscisic acid (ABA) signaling; mutations in this gene lead to dwarfism and reduced cell size [23]. The MADS-box transcription factor *ZmMADS42* exhibits dual regulatory functions: Its overexpression promotes early flowering while suppressing PH [24]. Furthermore, major QTL mapping identified qPH3.1 as a locus controlling PH. The candidate gene *ZmGA3ox2* encodes a key enzyme in gibberellin synthesis and regulates internode elongation by controlling active GA levels. Natural variation in its promoter is strongly correlated with PH, and loss-of-function mutants display dwarf phenotypes, highlighting its importance for molecular design of maize architecture [8].

Among the known genes involved in maize PH and EH regulation through gibberellin (GA) synthesis and signaling pathways are Anther ear1 (*AN1*), Dwarf3 (*D3*), Knotted1 (*KN1*), and DELLA-like protein genes [25,26,27]. The RS2 protein encoded by Rough Sheath2 (*RS2*) functions as a negative regulator of KNOX genes, and mutations in *RS2* result in dwarf phenotypes [28]. Viviparous8 (*VP8*) regulates abscisic acid (ABA) accumulation and is associated with reduced PH [29]. As maize yield improvement requires increasing planting density, high-density cultivation often results in thinner stems and reduced lodging resistance. Appropriate reduction of PH and EH can effectively enhance lodging resistance under such conditions. Therefore, elucidating the genetic architecture of PH and EH is essential for developing new maize varieties with optimally reduced PH and EH, representing a key objective in modern maize breeding [30,31].

Although previous studies have made significant progress, most GWASs have been conducted using a single year or a single population, leaving the environmental stability and genetic background specificity of the identified loci largely unclear. In this study, GWAS was performed for PH and EH using a natural population consisting of 479 maize inbred lines widely used in Northeast China, based on the mean phenotypic values from two years and genotypes from the maize 6H-60K SNP array. Twenty loci significantly associated with PH and eight loci significantly associated with EH were identified. From maize databases, candidate genes annotated within 100 kb upstream and downstream of each significant locus were retrieved, yielding 23 functionally annotated genes. These genes are predicted to function in processes such as ATP binding, symporter activity, zinc ion binding, and DNA binding, thereby providing a stronger foundation for understanding the genetic determinants of maize phenotypic traits.

## 2. Results

### 2.1. Descriptive Statistics of Phenotypic Traits

**Figure 1:**
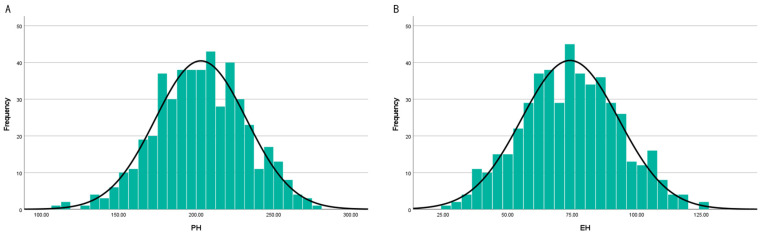
Normal distribution of PH and EH in 479 maize inbred lines: (**A**) frequency distribution of PH; (**B**) frequency distribution of EH.

Based on the original two-year dataset with two replications per year, broad-sense heritability (H^2^) was estimated using a mixed linear model to evaluate the genetic reliability of the phenotypic data. In this model, genotype, year, and the genotype-by-year interaction were treated as random effects, while replication nested within year was included as a random blocking factor. Heritability estimates were 0.793 for PH and 0.519 for EH, indicating that PH is under strong genetic control, whereas EH is moderately influenced by genetic factors. These results support the use of two-year average values in all subsequent analyses. Descriptive statistical analysis (Table 1 and Figure 1) showed that the skewness and kurtosis values for PH and EH traits ranged between −1 and 1, indicating that the phenotypic data followed a normal distribution. This distribution pattern is consistent with quantitative genetic traits and supports the suitability of the dataset for subsequent analyses.

The coefficient of variation (CV) for PH was 14.54%, indicating relatively low phenotypic variability, whereas EH exhibited a higher CV of 25.42%, reflecting greater variation among the inbred lines. The Shannon–Weaver diversity index was 1.172 for PH and 1.517 for EH, suggesting comparatively higher phenotypic diversity in EH.

### 2.2. Correlation Analysis Between Phenotypic Traits and Weighted Membership Function D-Values

The weighted membership function method was applied to calculate weighting coefficients for PH-related traits in the 479 maize inbred lines based on PH and EH phenotypic data. A higher weighting coefficient indicates greater relative contribution of a given trait. The calculated weighting coefficients were 0.43 for PH and 0.57 for EH, indicating a relatively greater contribution of EH.

**Figure 2:**
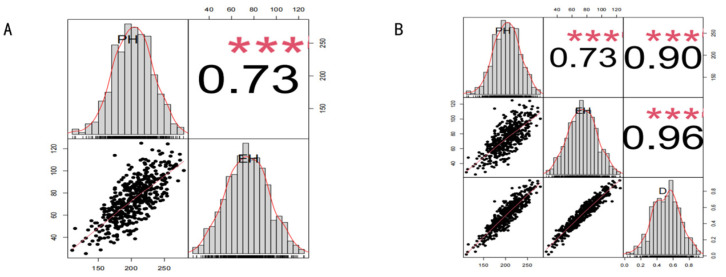
Frequency distributions and correlation analysis among PH, EH, and D-values in 479 maize inbred lines. (**A**) PH and EH; (**B**) PH, EH, and D-values. Frequency distribution histograms are displayed along the diagonal. Scatter plots of pairwise trait comparisons are shown below the diagonal, and correlation coefficients are presented above the diagonal. *** *p* < 0.001.

As illustrated in Figure 2, correlation analysis revealed strong associations between the measured traits and their corresponding weighted membership function values (D-values), with correlation coefficients (r) ranging from 0.73 to 0.96 (*p* < 0.001). All correlations were positive and highly significant (*p* < 0.001). Among them, the association between D-values and EH was the strongest, with a correlation coefficient of 0.96 (*p* < 0.001).

### 2.3. Cluster Analysis

**Figure 3:**
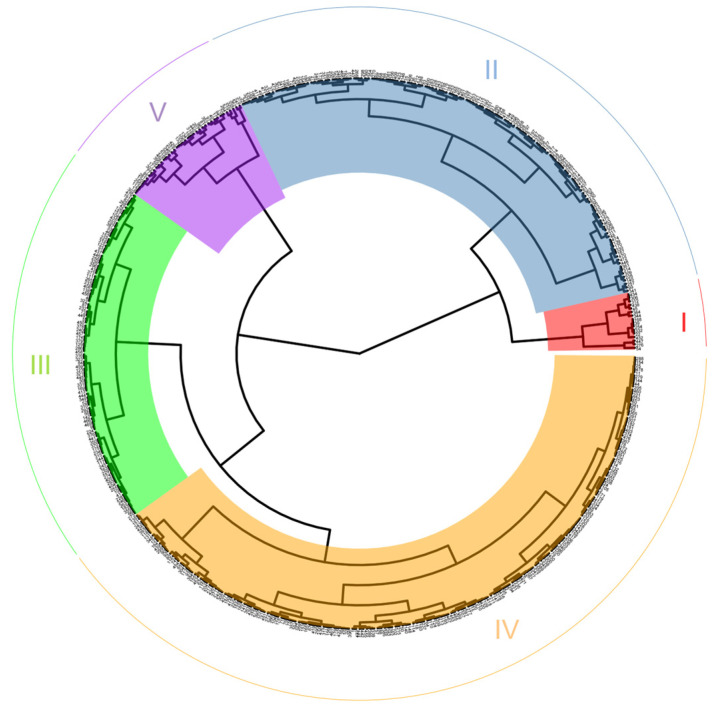
Hierarchical clustering of PH and EH traits in 479 maize inbred lines.

Cluster analysis was performed based on D-values, and the 479 maize inbred lines were classified into five joint categories based on both PH and EH (Figure 3).

Group I comprised 16 inbred lines (e.g., KL45 and Huotanghuang 17), characterized by relatively short plant stature, with PHs of approximately 130 cm and EHs around 30 cm. Group II included 137 inbred lines (e.g., G439 and KS23), representing a medium-stature group compared with Group I, with average PHs of approximately 170 cm and EHs of about 60 cm. Group III consisted of 95 inbred lines (e.g., G249 and Suixi 707), with PHs around 200 cm and EHs of approximately 70 cm. Group IV contained 192 inbred lines (e.g., 196 and Han 23), also classified as medium-to-tall types similar to Group III, exhibiting PHs of approximately 220 cm and EHs near 80 cm. Group V comprised 39 inbred lines (e.g., Ye52106 and B84), representing tall-stalked varieties with PHs of approximately 260 cm and ear positions around 110 cm above ground level.

### 2.4. Genetic Population Structure and Heterotic Group Assignment

**Figure 4:**
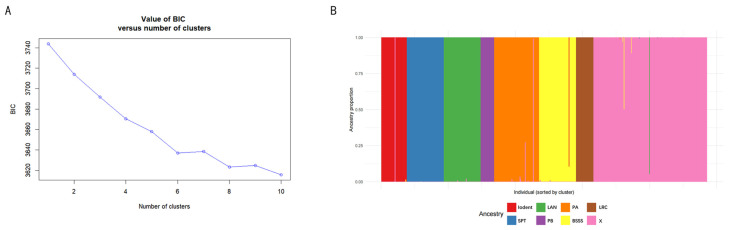
Population structure analysis of maize inbred lines based on DAPC. (**A**) Selection of the optimal K value; (**B**) Bar plot of population structure for the inbred lines.

DAPC analysis partitioned the 485 maize inbred lines into eight genetic clusters (Figure 4A). The BIC curve first declined at K = 6, increased at K = 7, dropped markedly at K = 8, rose again at K = 9, and decreased slightly at K = 10. Although the BIC value at K = 10 was slightly lower than that at K = 8, the reduction was minimal. Considering the prior assignment of the six tester lines and the biological relevance of K = 8 (i.e., distinguishing the Lancaster subgroup and identifying the X group), K = 8 was selected (Figure 4B). Based on posterior probabilities (>0.80), all six testers were correctly assigned to their expected heterotic groups, confirming the reliability of the analysis.

The 485 accessions were unequivocally divided into eight clusters (Supplementary Table S1). Cluster 1 (Iodent) comprised 38 lines, with representative inbreds such as PHPMO. Cluster 2 (Tangsipingtou) contained 55 lines, represented by Huangzao 4. Cluster 3 (Lancaster) included 55 lines, represented by Mo17. Cluster 4 (PB) consisted of 20 lines, represented by Qi319. Cluster 5 (PA) comprised 66 lines, represented by Ye478. Cluster 6 (Reid) contained 56 lines, represented by B73. Cluster 7 (Lancaster subgroup) included 26 lines; although sharing the same Lancaster background as Cluster 3, it formed a genetically distinct subgroup, possibly reflecting different breeding trajectories. Cluster 8 (X group) comprised 169 lines, represented by Jing724, representing a unique germplasm developed in northern China maize breeding. Notably, both Clusters 3 and 7 originated from the Lancaster background, but DAPC clearly separated them, indicating significant genetic differentiation within the Lancaster group. Taken together, the K = 8 solution is of clear genetic relevance and effectively resolves the population structure of the maize inbred panel.

### 2.5. Genome-Wide Association Study

GWAS for PH and EH was conducted using the MLM implemented in Tassel 5.0 software, based on 7861 high-quality SNP markers from 479 maize inbred lines.

**Figure 5:**
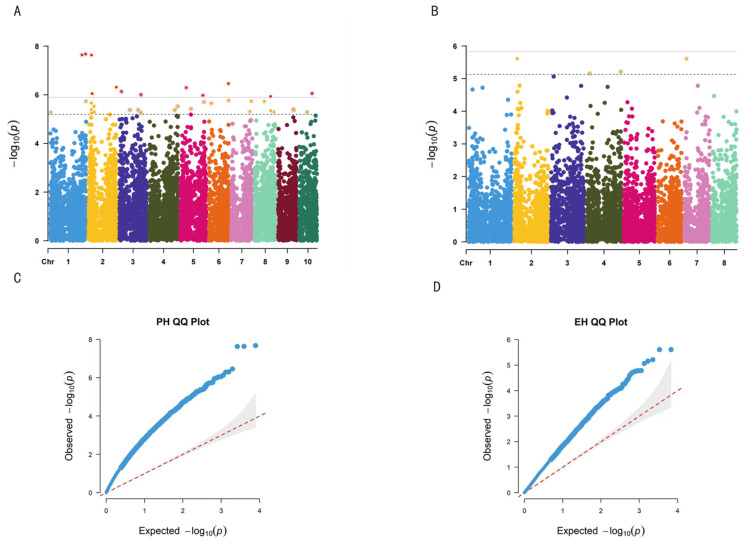
Association results for PH and EH. (**A**) Manhattan plots of PH; (**B**) Manhattan plots of PH EH; (**C**) QQ plot for PH; (**D**) QQ plot for EH.

Stature-associated loci: A total of 20 SNPs significantly associated with PH (*p* < 1.0 × 10^−5^) were identified on chromosomes 2, 4, 5, 6, 7, and 8 (Table 2, Figure 5). The most significant loci included SNP29894335 on chromosome 2 (*p* = 8.95 × 10^−7^), SNP165195376 on chromosome 6 (*p* = 3.48 × 10^−7^), and SNP189252058 on chromosome 5 (*p* = 1.06 × 10^−6^). No significant loci associated with PH were detected on chromosomes 1, 9, or 10.

A total of eight SNPs significantly associated with EH were identified, located on chromosomes 2, 3, 4, and 7. Chromosome 2 had the highest number of loci (three), followed by chromosomes 4 and 7 with two each, and chromosome 3 with one. Notably, SNP237681122 on chromosome 4 was significantly associated with both PH and EH (*p* = 7.43 × 10^−6^), suggesting potential pleiotropic effects at this locus. Compared with PH, the smaller number of significant loci and relatively higher *p*-values for EH indicate that EH may be regulated by a more complex genetic architecture or may exhibit greater sensitivity to environmental variation.

### 2.6. Identification of Plant Architecture-Associated Candidate Genes

Based on the LD decay distance of the population, a 100-kb flanking region was defined upstream and downstream of each significant SNP for candidate gene identification. Within these ±100 kb intervals surrounding the 28 significant loci associated with the two height traits, 78 candidate genes were identified, 23 of which had functional annotations.

Among them, some SNPs were significantly associated with both PH and EH (co-localized SNPs), such as SNP Affx-291378242 at position 237,681,122 bp on chromosome 4. No known functional genes were annotated within the linkage disequilibrium intervals containing these co-localized SNPs; therefore, they were not included as candidate genes. By integrating functional annotation information for all genes within each locus and examining their expression patterns across different tissues of B73, the most likely candidate gene for each locus was determined. The selected candidate genes and their corresponding annotations are presented in Table 3.

### 2.7. Functional Prediction of Candidate Genes

**Figure 6:**
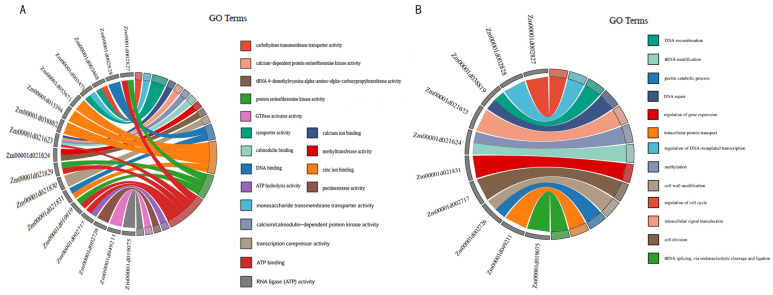
GO enrichment analysis was performed on candidate genes. Genes sharing identical colors are grouped according to distinct GO annotations. (**A**) Molecular function enrichment analysis; (**B**) Biological process enrichment analysis.

The 23 identified candidate genes were enriched in a total of 31 GO terms (Figure 6; Table 4 and Table 5). These GO terms were classified into two major functional categories.

The first category comprised 13 terms related to biological processes. Representative functions included zinc ion binding associated with *Zm00001d015394* (GO:0008270), ATP binding associated with *Zm00001d021829* (GO:0005524), and pectinesterase activity associated with *Zm00001d002726* (GO:0030599), among others.

The second category included 18 terms corresponding to molecular functions. These encompassed DNA repair associated with *Zm00001d038819* (GO:0006281), methylation associated with *Zm00001d021624* (GO:0032259), and cell wall modification (GO:0042545).

### 2.8. Analysis of Basic Characteristics of the Candidate Genes

For each significant SNP, a 100-kb flanking region upstream and downstream was designated as the candidate interval based on the population’s linkage disequilibrium (LD) decay distance. Surrounding the 28 significant loci, these ±100 kb intervals yielded a total of 78 candidate genes, 23 of which had functional annotations. By integrating functional annotation information and examining expression patterns across different tissues of B73, the most likely candidate gene for each locus was determined. Among these, *Zm00001d002726* and *Zm00001d015394* were selected for further analysis because they are located near significant SNPs, possess well-defined functional domains, and fall within known QTL regions.

**Figure 7:**
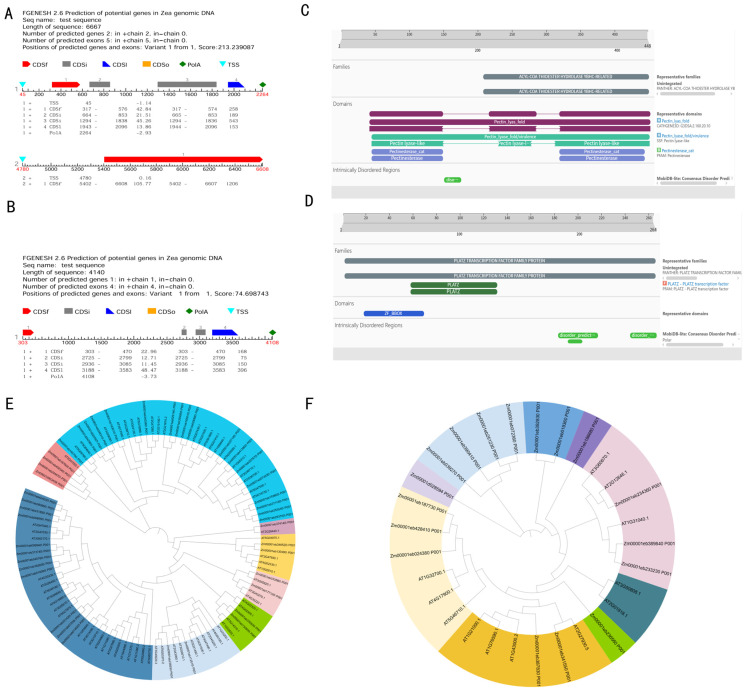
Basic characteristics of two potential candidate genes. (**A**,**B**) Gene structure; (**C**,**D**) conserved protein domain analysis; (**E**) Pectin methylesterase gene family; (**F**) PLATZ transcription factor family.

The *Zm00001d002726* gene is located in the bin 2.04 region on maize chromosome 2 and spans 6667 bp in full length. It contains four exons, with a coding sequence (CDS) of 1143 bp encoding a stable basic protein of 448 amino acids with moderate hydrophobicity (Supplementary Table S2) (Figure 7). Conserved domain analysis of the *Zm00001d002726* protein indicates that it functions as a typical plant cell wall metabolism-related protein, promoting cell wall modification and participating in pectin catabolism.

Transmembrane structure analysis of *Zm00001d002726* (Supplementary Figure S1) revealed zero predicted transmembrane helices, indicating that it is a non-transmembrane protein. The protein is predicted to localize extracellularly. Expression analysis suggests that *Zm00001d002726* may play a regulatory role in PH and ear position, with moderate expression levels indicative of a fundamental regulatory function. Phylogenetic analysis showed that the protein encoded by *Zm00001eb074530* shares the highest homology with *Zm00001eb307670* in maize and *AT1G69940* in *Arabidopsis* (Figure 7E).

The *Zm00001d015394* gene is mapped to the bin5.05 region on maize chromosome 5 and spans 4140 bp in full length. It comprises four exons, with a coding sequence (CDS) of 789 bp encoding a stable basic protein of 264 amino acids with moderate hydrophobicity (Supplementary Table S3). Conserved domain prediction indicates that the *Zm00001d015394* protein belongs to the PLATZ transcription factor family, a group of transcription factors specific to plants. The protein contains two overlapping PLATZ domains and a ZF_BBOX zinc finger domain, which together likely enhance its functional activity. Molecular function classification highlights zinc ion binding activity (GO:0008270), consistent with the presence of the ZF_BBOX zinc finger domain and supporting its role in transcriptional regulation.

Transmembrane domain analysis demonstrated that *Zm00001d015394* lacks transmembrane domains and is therefore also classified as a non-transmembrane protein. Expression analysis indicates that *Zm00001d015394* exerts strong regulatory effects on PH and ear position (Supplementary Figure S2). Phylogenetic analysis revealed that the protein encoded by *Zm00001eb233230* exhibits the highest homology with *Zm00001eb389840* in maize and *AT1G31040* in *Arabidopsis* (Figure 7F).

**Figure 8:**
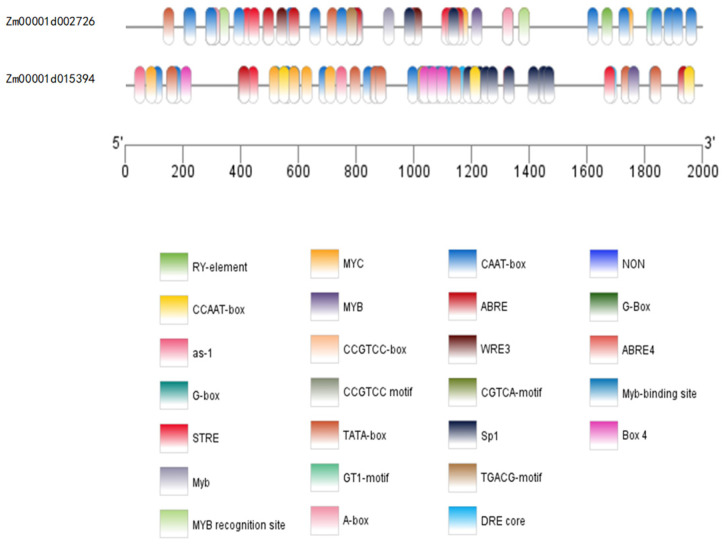
Analysis of cis-acting regulatory elements in the promoter regions of candidate genes.

Both genes contain core promoter elements (TATA-box, CAAT-box), light-responsive elements (G-box, GT1-motif), abiotic stress-responsive elements (ABRE, STRE, DRE core), and transcription factor binding sites such as MYB and MYC (Figure 8). These features suggest that they function as central regulatory genes in plant growth and development.

## 3. Discussion

### 3.1. Significance of Phenotypic Diversity in Regional Maize Breeding Materials

Extensive research has demonstrated that PH and EH are quantitative traits rather than simple Mendelian characteristics, exhibiting continuous variation controlled by multiple genetic loci and influenced by environmental factors [32,33]. For the genetic architecture of PH, Peiffer utilized a nested association mapping (NAM) population consisting of thousands of inbred lines to evaluate PH and EH across 13 environments, revealing that PH exhibits a heritability exceeding 90% and is characterized by highly polygenic inheritance [34].

The genetic diversity of PH and EH within global and regional germplasm collections constitutes a fundamental resource for breeding improvement. Germplasm banks preserve extensive variation in architectural traits, providing essential materials for allele mining, parental selection, and trait optimization [35]. Such diversity offers valuable phenotypic materials for elucidating the genetic regulatory mechanisms underlying PH and EH. As central components of maize plant architecture, PH and EH are among the key determinants of yield performance. A well-balanced plant architecture is therefore essential for achieving stable yield enhancement [16].

In the present GWAS analysis of maize PH in Northeast and North China, the selected population materials reflect regional breeding practices. These included widely used backbone inbred lines such as Dong 237, Ji 853, and Dan 598, as well as lines introduced and subsequently adapted through localization, including PH6WC, Mo17Ht, and PH09B. The population also incorporated inbred lines developed for specific ecological regions, such as Jing92 and Jing501. This composition provided substantial phenotypic variation in PH while ensuring direct relevance to regional breeding objectives. The phenotypic performance of these materials further confirmed their representativeness.

Broad-sense heritability was estimated using raw data from two replicates per year over two years to assess the genetic reliability of the phenotypic measurements. The estimates were 0.793 for PH and 0.519 for EH, suggesting that the former is under strong genetic control while the latter is moderately influenced by genetic factors. Field phenotypic evaluation showed that PH in the experimental materials exhibited a continuous and approximately normal distribution, ranging from 110 to 279 cm. The relatively shorter inbred line Dong 237 (179 cm) displayed clear phenotypic differentiation compared with taller lines such as PH09B (254 cm) and PH6WC (227 cm). Genetic structure analysis partitioned the inbred lines into eight genetic clusters, which not only validated the prior assignments of the tester lines but also revealed genetic differentiation within the Lancaster group and the distinctiveness of the northern China X group, offering clear genetic and breeding implications. These differences reflect both underlying genetic variation at height-related loci and selection strategies aligned with regional breeding goals in Northeast China, namely early maturity, low-temperature tolerance, high planting density, enhanced lodging resistance, and suitability for mechanical harvesting.

### 3.2. SNP Loci Associated with PH and EH Identified Through Genome-Wide Association Study

GWAS and QTL mapping are powerful approaches for dissecting the complex genetic architecture of maize PH and EH. Wang et al. (2025) conducted GWAS analysis of maize inbred lines from Southeast China using the Maize6H-60K SNP array and identified 40 significant SNPs, including nine associated with PH and 16 with EH [16]. The phenotypic variation explained ranged from 3.42% to 25.92% for PH and from 2.49% to 38.49% for EH. Five stable SNPs were identified. In addition, the SNP (AX-247241325) located at 177,277,477 bp on chromosome 5 identified in that study and the PH-associated SNP Affx-291446353 located at 196,854,197 bp on chromosome 5 identified in the present study both map to the bin 5.06 interval. Our results, together with previous reports, confirm that both traits are highly polygenic in nature [36,37,38]. Several genomic regions, including bin 1.05, 5.04/05, and 6.04/05, have been repeatedly identified as harboring QTLs associated with PH and EH across different populations and analytical methods [39]. In particular, the bin 5.05–5.07 region on chromosome 5 has been consistently reported as a major locus, frequently containing co-localized QTLs for both PH and EH [36]. The fine-mapping and cloning of a causative rare SNP within the Brachytic2 (*Br2*) gene in this region illustrate the effectiveness of integrating high-resolution mapping with functional validation to progress from QTL identification to gene characterization [40]. Moreover, the integration of multi-omics datasets—including genomics, transcriptomics, and phenomics—across diverse genetic backgrounds and environments will be essential for elucidating gene regulatory networks and clarifying genotype-by-environment interactions that ultimately shape plant architecture [41].

In recent years, GWAS has emerged as a rapid and efficient strategy for systematically identifying the genetic components underlying complex plant traits [42,43,44]. In the present study, a natural population comprising 479 maize inbred lines with diverse genetic backgrounds was used for genome-wide association analysis, resulting in the identification of 28 significant SNP markers. The PH-associated SNP Affx-291446353, located at 196,854,197 bp on chromosome 5, falls within the bin 5.06 interval previously reported by Zhou et al. [45]. The EH-associated marker Affx-159160203 is located within the bin 3.05 segment, corresponding to the major EH QTL mapped by Pan et al. [46]. Additionally, the PH-associated SNP Affx-291446353 co-localizes within the bin 5.06–5.07 region identified as a major QTL region for PH and EH by Xin Li et al. [47].

Notably, SNP Affx-291378242 located at 237,681,122 bp on chromosome 4 in this study was significantly associated with both traits (PH: *p* = 7.43 × 10^−6^; EH: *p* = 6.14 × 10^−6^), suggesting that this locus may harbor genetic variants underlying the common genetic basis of PH and EH. The presence of such co-localized SNPs indicates that PH and EH are not genetically independent but may share certain regulatory pathways or exhibit pleiotropy. However, because no functionally characterized genes were annotated within the linkage disequilibrium interval containing this co-localized SNP, it was not included as a candidate gene for further analysis.

Allelic effect analysis of the co-localized SNPs demonstrated that all 28 loci identified in this study exhibited significant allelic variation associated with distinct phenotypic differences, which is consistent with the trend of higher heritability for PH (0.793) than for EH (0.519), supporting the robustness of the association results. These co-localized SNPs therefore represent valuable targets for the development of molecular markers for the genetic improvement of PH and EH.

### 3.3. Fundamental Characteristics of Genes Related to PH and EH

This study identified 23 candidate genes potentially associated with PH and EH. Among these, SNP88083460 was associated with *Zm00001d015394* (PLATZ-transcription factor 8), a member of the plant-specific AT-rich sequence and zinc-binding (PLATZ) protein family. Meanwhile, SNP22657683 was associated with *Zm00001d002726*, corresponding to Pectin Methylesterase 36.

PH is determined by the rate of cell growth, which depends largely on the extent of cell wall elongation [48]. Gene structure analysis revealed that both *Zm00001d002726* and *Zm00001d015394* contain four exons, a structural feature consistent with previously characterized height-related genes [49]. Conserved domain analysis provided insight into their potential functions. *Zm00001d002726* encodes a pectinesterase, a classical pectin-modifying enzyme likely involved in pectin degradation and remodeling within the cell wall. By altering cell wall pectin structure, this enzyme may regulate wall extensibility and thereby influence cell elongation. In contrast, *Zm00001d015394* encodes a PLATZ family transcription factor containing a conserved zinc finger domain, suggesting its ability to bind specific DNA sequences and regulate transcription of downstream target genes [50,51]. This regulatory mode is comparable to that of other transcription factors (e.g., WRKY and MYB) known to influence PH by modulating hormone-related pathways such as gibberellin and auxin signaling [52].

Transmembrane domain prediction indicated that neither protein contains transmembrane helices, suggesting both are non-transmembrane proteins. Promoter cis-element analysis of the 2000 bp upstream regions identified core promoter elements (TATA-box and CAAT-box), as well as regulatory elements including G-box (light and hormone response element), CGTCA-motif and TGACG-motif (jasmonic acid response elements), and ABRE (ABA response element). Detection also revealed binding sites for transcription factors such as MYB and MYC. These results align with the GO enrichment analysis, which highlighted molecular functions including zinc ion binding and pectinesterase activity, along with biological processes such as pectin catabolism and cell wall modification [53,54]. Moreover, given that light signaling and gibberellin signaling often act synergistically in regulating internode elongation [55], the presence of stress- and hormone-responsive elements suggests that these genes may participate in the adaptive regulation of PH under varying environmental conditions.

Functional studies of PLATZ and PME family members in model plants further support the predicted roles of *Zm00001d015394* and *Zm00001d002726*. In *Arabidopsis*, *PLATZ1* and *PLATZ2* are involved in seed desiccation tolerance and vegetative drought tolerance [56]; the activation-tagged mutant *ore15-1D* exhibits enlarged leaves and extended leaf longevity, and ORE15 promotes leaf growth while suppressing senescence by modulating the GRF/GRF-interacting factor pathway, as well as regulating root apical meristem size through auxin–cytokinin crosstalk [57]. Grain development and cell proliferation in rice are regulated by the PLATZ gene *GL6* [58]. These findings suggest that PLATZ transcription factors, including *Zm00001d015394*, may participate in cell elongation and organ growth through diverse regulatory mechanisms.

Similarly, the pectin methylesterase (PME) family has been systematically characterized in plants [59]. PME activity, modulated by local pH, can either loosen or rigidify the cell wall, thereby influencing cell elongation and plant growth [60,61]. Different PME members exhibit tissue-specific expression patterns during stem elongation [60,62], root development [61], and fruit ripening [63,64], highlighting their close association with cell wall remodeling and internode elongation. These functional attributes support the predicted role of *Zm00001d002726* in cell wall modification and PH regulation.

Bioinformatics analyses further support that *Zm00001d015394* may function through zinc ion binding activity, whereas *Zm00001d002726* likely participates in cell wall modification through pectinesterase activity. These results are consistent with the GO enrichment analysis.

In summary, based on GWAS analysis of PH and EH in core maize inbred lines from the East-Northeast China region, this study clarifies the genetic architecture and key regulatory loci underlying these traits and provides candidate genes with predicted functional relevance. Based on GWAS mapping results and functional predictions of candidate genes, *Zm00001d015394* and *Zm00001d002726* represent promising targets for improving maize plant architecture and for breeding varieties with improved tolerance to high-density planting and environmental stress. These candidate genes provide valuable targets for functional characterization, and future studies using CRISPR/Cas9 or overexpression experiments are needed to confirm their precise roles in the regulation of PH and EH.

## 4. Materials and Methods

### 4.1. Experimental Materials and Field Design

A total of 479 elite inbred lines from Harbin, China were used in this study (Supplementary Table S4). These comprised 98 exchange lines obtained from northern Chinese maize breeding institutions; 358 domestic maize inbred lines with diverse pedigrees provided by Researcher Li Xinhai from the Institute of Crop Science, Chinese Academy of Agricultural Sciences; and 23 superior maize inbred lines independently developed by Northeast Agricultural University.

Field trials were carried out in 2023 and 2024 at the Experimental Training Base of Northeast Agricultural University, located in Xiangyang Township, Xiangfang District, Harbin (45.7° N, 126.9° E). Each inbred line was grown in two rows of 3 m length, with a row spacing of 0.65 m and a plant spacing of 0.25 m, which is the conventional density for maize inbred line trials in Northeast China. Field management was carried out according to standard agronomic practices [65].

### 4.2. Trait Measurement and Phenotypic Data Analysis

Field investigations of PH and EH were conducted at the onset of physiological maturity. To minimize border effects, the first plant in each row was excluded, and three plants per row were randomly selected for measurement. PH was defined as the vertical distance from the base at ground level to the apex of the tassel (cm). EH was measured as the vertical distance from the base at ground level to the first node bearing the ear (cm) [66]. SPSS 27.0 software was used to assess the normality of the phenotypic data [6], and coefficients of variation were calculated. Genetic diversity analysis was performed using Excel. The weight coefficients and D-value were calculated using the membership function method. Correlation analysis and cluster analysis were performed using R (version 4.4.3) software, and the corresponding graphical outputs were generated. Except for the estimation of heritability, which was based on the two-year data with two replications, all remaining analyses and calculations were performed using the mean values. To estimate broad-sense heritability and dissect the genetic and environmental effects on phenotypic variation, a mixed linear model was constructed using the lme4 package (version 1.1-35) in R software (version 4.4.3). Shannon diversity indices (H’) were calculated in Excel [67].

Random effects in the model were fitted for genotype (LINE), year (YEAR), and their interaction (LINE:YEAR), with replication nested within year (YEAR:REP) serving as a random block effect. The model is expressed as:

Y_ijkl_ = μ + G_i_ + Y_j_ + (GY)_ij_ + R_k(j)_ + ε_ijkl_

In this model, Y_ijkl_ denotes the phenotypic value for the _i_-th genotype in the _j_-th year, k-th replication, and l-th observation; μ is the overall mean; G_i_ represents the random effect of genotype; Y_j_ is the random effect of year; (GY)_ij_ is the random effect of the genotype-by-year interaction; R_k(j)_ is the random effect of replication nested within year; and ε_ijkl_ is the residual error.

Using the variance components estimated from this model, broad-sense heritability (H^2^) was derived for each trait. Given the experimental layout with two years and two replications per year, heritability was calculated as:

H^2^ = σ^2^_G_/σ^2^_G_ + σ^2^_G_ × _Y_/n_Y_ + σ^2^_e_/n_Y_ × n_R_

where σ2G is the genetic variance, σ2G × Y is the genotype and year interaction variance, σ2e is the residual variance, nY is the number of years (2), and nR is the number of replications (2). All variance components were estimated using the restricted maximum likelihood (REML) method [68].

The Shannon–Wiener index was calculated as:

H’ = −∑ (pᵢ ln pᵢ)

where H’ represents the diversity index of a given trait, and pᵢ = Nᵢ/N denotes the frequency of occurrence of the iᵗʰ phenotypic class, with Nᵢ representing the number of individuals in the iᵗʰ class and N representing the total number of individuals [16].

We calculated the weighted membership function value (D value) using the mean values of each trait. The formulas for this method are as follows:

μ(X_i_) = (X_i_ − X_i_min)/(X_i_max − X_i_min)

W_i_ = CV_i_/_i_ = 1nCV_i_(_i_ = 1, 2, 3, … n)

D = _i_ = 1n[μ(X_i_) ∙ W_i_](_i_ = 1, 2, 3, … n)

where: μ(X_i_) is the membership function value of each tested inbred line for trait;

Xi is the mean value of the related trait for each tested inbred line;

Ximax and Ximin are the maximum and minimum values of X_i_ among the tested

inbred lines, respectively;

W_i_ is the weighting coefficient for the _i_-th trait;

CV_i_ is the coefficient of variation for μ(X_i_) among the tested inbred lines;

D is the weighted membership function value.

### 4.3. DNA Extraction and Genotyping

Genomic DNA was extracted from leaf tissue using the CTAB method, and DNA quality was evaluated prior to genotyping. Genotyping was performed using the Maize6H-60K chip [6], developed by the Maize Research Centre in Beijing. Raw genotype data were initially processed using TASSEL 5.0 software. After quality control filtering with criteria of minor allele frequency (MAF) > 0.01 and missing rate < 0.01, a total of 7861 high-quality SNPs were retained for subsequent analyses [69].

### 4.4. Population Structure Analysis

A total of 485 maize inbred lines, comprising 479 core accessions and six tester lines representing distinct heterotic groups, were subjected to population structure analysis. The analysis was performed using the adegenet package (v2.1.11) in R [70]. A genind object was constructed from the SNP genotype data, and missing values were imputed with the mean. Principal component analysis (PCA) was conducted, and the first 100 principal components (explaining >95% of the cumulative variance) were retained. Sequential K-means clustering on the PCA-transformed data was carried out using the find.clusters function, and the Bayesian Information Criterion (BIC) was employed to evaluate model fit for K values ranging from 2 to 10. The optimal number of clusters was determined by combining the BIC trend with the prior assignment of the tester lines. Finally, Discriminant Analysis of Principal Components (DAPC) was applied to obtain soft clustering [71], yielding posterior membership probabilities for each individual. Each line was assigned to the genetic cluster with the highest posterior probability (threshold > 0.80).

### 4.5. Genome-Wide Association Analysis of Plant Height and Ear Height in Maize Germplasm

GWAS was conducted between SNP markers and the two-year arithmetic means of PH and EH for the 479 accessions using the MLM implemented in Tassel 5.0 software [72,73]. The significance threshold for the F-test was determined through permutation testing with 1000 permutations [74].

Associations between SNP markers and phenotypic traits were considered significant when *p*-values < 1 × 10^−4^ (−log_10_ *p* > 4). The association results were visualized using Manhattan plots and QQ plots generated with the “CMplot” package in R [75].

### 4.6. Candidate Gene Prediction

Candidate gene prediction was conducted based on the B73 reference genome (B73_RefGen_v4) obtained from MaizeGDB. Significant SNP markers were used as anchor points, and flanking regions extending 100 kb upstream and downstream were defined according to the extent of linkage disequilibrium decay [76]. All genes located within these intervals were retrieved from the maize database MaizeGDB (http://www.maizegdb.org) (accessed on 5 December 2025). Functional annotation of candidate genes was performed by integrating information from MaizeGDB and NCBI (https://www.ncbi.nlm.nih.gov) (accessed on 6 December 2025).

### 4.7. Procedure for Candidate Gene Identification and Annotation

GO enrichment analysis of the candidate genes was conducted using the UniProt database (https://www.uniprot.org/), and KEGG pathway enrichment analysis was performed through the Phytozome database (https://phytozome-next.jgi.doe.gov/) (accessed on 7 December 2025) [77,78]. Functional annotation of the candidate genes was based on information retrieved from NCBI (https://www.ncbi.nlm.nih.gov/) and MaizeGDB. The Chord Map was generated using an online bioinformatics visualization platform (http://www.bioinformatics.com.cn) (accessed on 9 December 2025) [66].

### 4.8. Characterizing the Basic Features of Candidate Genes

Characterization of candidate genes was performed using multiple public databases and online tools. Gene-related information was retrieved from Phytozome (https://phytozome-next.jgi.doe.gov/) (accessed on 8 December 2025), and gene structure prediction was conducted using the Softberry online platform (http://www.softberry.com/) (accessed on 10 December 2025). The physicochemical properties of the encoded proteins were analyzed using EXPASY (https://web.expasy.org/protparam/) (accessed on 11 December 2025). Promoter cis-acting elements were examined through the PlantCare platform (https://bioinformatics.psb.ugent.be/webtools/plantcare/html/) (accessed on 5 December 2025). Conserved domains of the encoded proteins were identified using InterPro (https://www.ebi.ac.uk/interpro/search/sequence/) (accessed on 6 December 2025). Transmembrane domains were predicted using the TMHMM-2.0 online tool (https://services.healthtech.dtu.dk/service.php?TMHMM-2.0) (accessed on 7 December 2025). Spatiotemporal expression patterns of the candidate genes were analyzed using Gene Expression And Protein Tools (https://bar.utoronto.ca/#GeneExpressionAndProteinTools) (accessed on 8 December 2025). Protein sequences with the highest similarity were identified by BLAST P in NCBI using the encoded protein sequences, and a phylogenetic tree was constructed using MEGA X (version 10.2.7).

## 5. Conclusions

This study applied GWAS to a panel of 479 maize inbred lines from Northeast and North China. After controlling for relatedness, the MLM identified 20 loci associated with PH on chromosomes 2, 4, 5, 6, 7, and 8, and 8 loci associated with EH on chromosomes 2, 3, 4, and 7. A total of 23 candidate genes were identified, among which five are potentially involved in regulating plant architecture, including pectin methylesterase 36 and the PLATZ8 transcription factor. GO enrichment analysis classified these genes into 18 biological processes and 13 molecular functions. Structural analysis indicated that both *Zm00001d002726* and *Zm00001d015394* share a four-exon configuration, reflecting a compact gene architecture similar to that of known PH-related genes. Conserved domain analysis suggested that *Zm00001d002726* is associated with pectin metabolism and may influence cell expansion through modification of cell wall pectin. In contrast, *Zm00001d015394* encodes a PLATZ family transcription factor containing a conserved zinc finger domain that confers DNA-binding capacity, suggesting its potential involvement in PH regulation. *Zm00001d002726*, which is associated with EH, may modulate the physical properties of the cell wall. Although these two genes regulate PH and EH, respectively, they are likely to function at different biological levels: *Zm00001d002726* may influence the physical properties of the cell wall, whereas *Zm00001d015394* may regulate gene expression programs. Overall, this study provides insight into the genetic and mechanistic basis of maize PH and EH and establishes a foundation for future variety improvement and molecular breeding.

## Figures and Tables

**Table 1:** Descriptive statistics of PH and EH in 479 maize inbred lines.

Trait	Average (cm)	Variance	H’	CV	Skewness	Kurtosis	Range (cm)
PH	203.01 ± 29.52	871.56	1.17	14.54%	−0.12 ± 0.11	−0.06 ± 0.22	110.67–279.33
EH	74.12 ± 18.84	355.09	1.52	25.42%	0.04 ± 0.11	−0.34 ± 0.22	25.33–125

**Table 2:** SNPs associated with PH and EH in 479 maize inbred lines.

Trait	SNP Marker	Chromosome	Position (bp) ^(1)^	*p*-Value ^(2)^	*R*^2^ (%) ^(3)^
PH	Affx-291405576	2	23,380,357	2.19 × 10^−6^	5.62
PH	Affx-291402125	2	23,937,292	4.11 × 10^−6^	5.34
PH	Affx-88980474	2	29,894,335	8.95 × 10^−7^	6.05
PH	Affx-291431544	2	45,316,415	2.98 × 10^−6^	5.51
PH	Affx-159069410	4	213,570,327	4.25 × 10^−6^	5.33
PH	Affx-88987729	4	229,716,096	7.32 × 10^−6^	5.09
PH	Affx-291378242	4	237,681,122	7.43 × 10^−6^	5.11
PH	Affx-291383305	4	238,603,204	2.99 × 10^−6^	5.51
PH	Affx-291383012	5	88,083,460	3.80 × 10^−6^	5.41
PH	Affx-291425032	5	189,252,058	1.06 × 10^−6^	5.97
PH	Affx-291446353	5	196,854,197	1.96 × 10^−6^	5.72
PH	Affx-291440309	6	21,376,772	2.25 × 10^−6^	5.77
PH	Affx-291418872	6	165,195,376	3.48 × 10^−7^	6.45
PH	Affx-158935629	6	166,379,753	1.72 × 10^−6^	5.72
PH	Affx-291441995	7	154,586,976	4.95 × 10^−6^	5.30
PH	Affx-159043453	7	158,308,107	1.20 × 10^−5^	4.92
PH	Affx-291416625	7	164,029,829	1.10 × 10^−5^	5.02
PH	Affx-159033068	7	167,880,829	1.83 × 10^−6^	5.70
PH	Affx-291425979	8	80,019,950	1.87 × 10^−6^	5.70
PH	Affx-291445873	8	133,309,877	1.16 × 10^−6^	5.92
EH	Affx-159017615	2	20,451,392	2.45 × 10^−6^	5.49
EH	Affx-159116552	2	22,657,683	2.51 × 10^−5^	4.50
EH	Affx-159130700	2	37,470,159	1.64 × 10^−5^	4.69
EH	Affx-159160203	3	19,262,449	8.66 × 10^−6^	4.92
EH	Affx-291445893	4	20,883,417	7.00 × 10^−6^	5.03
EH	Affx-291378242	4	237,681,122	6.14 × 10^−6^	5.09
EH	Affx-291400987	7	15,342,968	2.45 × 10^−6^	5.50
EH	Affx-291391542	7	93,417,110	1.65 × 10^−5^	4.64

^(1)^ Chromosome and base pair position indicate the location. ^(2)^ *p*-values are shown in scientific notation, with values below 10^−4^ corresponding to a 5% type I error. ^(3)^ *R*^2^, represents the proportion of phenotypic variation explained.

**Table 3:** Candidate genes associated with PH and EH: their loci and functional annotations.

Chromosome	Trait	Gene ID	SNP	Gene Function Annotation
2	PH	*Zm00001d002827*	SNP23937292	Serine/threonine protein kinase GRIK1
2	PH	*Zm00001d002828*	SNP23937292	NAC transcription factor 131
2	PH	*Zm00001d003468*	SNP45316415	sugar transport protein 10
2	PH	*Zm00001d003471*	SNP45316415	sugar transport protein 6
4	PH	*Zm00001d053671*	SNP238603204	zinc finger protein 11
5	PH	*Zm00001d015394*	SNP88083460	PLATZ transcription factor 8
6	PH	*Zm00001d038819*	SNP165195376	Non-structural maintenance of chromosomes element 4 homolog A
6	PH	*Zm00001d038882*	SNP166379753	Chromosome Condensation Regulator 4
7	PH	*Zm00001d021623*	SNP158308107	calcium dependent protein kinase 8
7	PH	*Zm00001d021624*	SNP158308107	tRNA wybutosine-synthesising protein 2/3/4
7	PH	*Zm00001d021829*	SNP164029829	symbiosis receptor-like kinase-like1
7	PH	*Zm00001d021830*	SNP164029829	symbiosis receptor-like kinase-like1
7	PH	*Zm00001d021831*	SNP164029829	C3H-transcription factor 32
8	PH	*Zm00001d010918*	SNP133309877	Maize nuclear envelope-associated protein 1
8	PH	*Zm00001d010919*	SNP133309877	pto kinase interactor 1
2	EH	*Zm00001d002717*	SNP22657683	cell division control protein 48 homolog C
2	EH	*Zm00001d002721*	SNP22657683	cortical cell-delineating protein
2	EH	*Zm00001d002723*	SNP22657683	Maize LINC KASH Grass-Specific 1
2	EH	*Zm00001d002726*	SNP22657683	pectin methylesterase 36
3	EH	*Zm00001d039911*	SNP19262449	Feronia-like receptor 17
3	EH	*Zm00001d039916*	SNP19262449	leucine-rich repeat/extensin-like chimera protein 14
7	EH	*Zm00001d019075*	SNP15342968	tRNA ligase 1

**Table 4:** Molecular function annotation (GO) analysis of candidate genes.

Gene ID	GO Codes	
*Zm00001d002827*	GO:0004674	GO:0005524
*Zm00001d002828*	GO:0003677	
*Zm00001d003468*	GO:0015293	GO:0015144
*Zm00001d003471*	GO:0015145	GO:0015293
*Zm00001d053671*	GO:0008270	
*Zm00001d015394*	GO:0008270	
*Zm00001d038882*	GO:0008270	
*Zm00001d021623*	GO:0005524	GO:0005509
*Zm00001d021623*	GO:0009931 GO:0004683 GO:0005516	
*Zm00001d021624*	GO:0008168	GO:0102522
*Zm00001d021829*	GO:0005524	GO:0004674
*Zm00001d021830*	GO:0003714	
*Zm00001d021831*	GO:0003677	GO:0008270
*Zm00001d010919*	GO:0005524	GO:0004674
*Zm00001d002717*	GO:0005524	GO:0016887
*Zm00001d002726*	GO:0030599	
*Zm00001d049211*	GO:000509	
*Zm00001d019075*	GO:0003972	

**Table 5:** Biological process annotation (GO) analysis of candidate genes.

Gene ID	GO Codes	
*Zm00001d002827*	GO:0051726	
*Zm00001d002828*	GO:0006355	
*Zm00001d038819*	GO:0006310	GO:0006281
*Zm00001d021623*	GO:0035556	
*Zm00001d021624*	GO:0032259	GO:0006400
*Zm00001d021831*	GO:0010468	
*Zm00001d002717*	GO:0051301	
*Zm00001d002726*	GO:0042545	GO:0045490
*Zm00001d049211*	GO:0006886	
*Zm00001d019075*	GO:0006388	

## Data Availability

The data presented in this study are available upon request from the corresponding author.

## Supplementary Materials

The following supporting information can be downloaded at: https://www.mdpi.com/article/10.3390/plants15091383/s1, Figure S1: Transmembrane Structure Analysis of Candidate Genes. Figure S2: Prediction of Spatiotemporal Expression Patterns of Candidate Genes. Table S1: 485 Maize Inbred Line Groups. Table S2: Physicochemical Properties of the *Zm00001d002726* Gene. Table S3: Physicochemical Properties of the *Zm00001d015394* Gene. Table S4: Sources of 479 elite inbred lines.
