# Supplementary material for: Genome-Wide Association Study of Plant and Ear Height in Maize (Zea mays L.) and Identification of Candidate Genes
Source: Plants (Basel). 2026 Apr 30;15(9):1383. doi: 10.3390/plants15091383 (PMC13165044; doi:10.3390/plants15091383)

**Supplementary Figure S1** Transmembrane Structure Analysis of Candidate Genes

**Supplementary Figure S2** Prediction of Spatiotemporal Expression Patterns of Candidate Genes


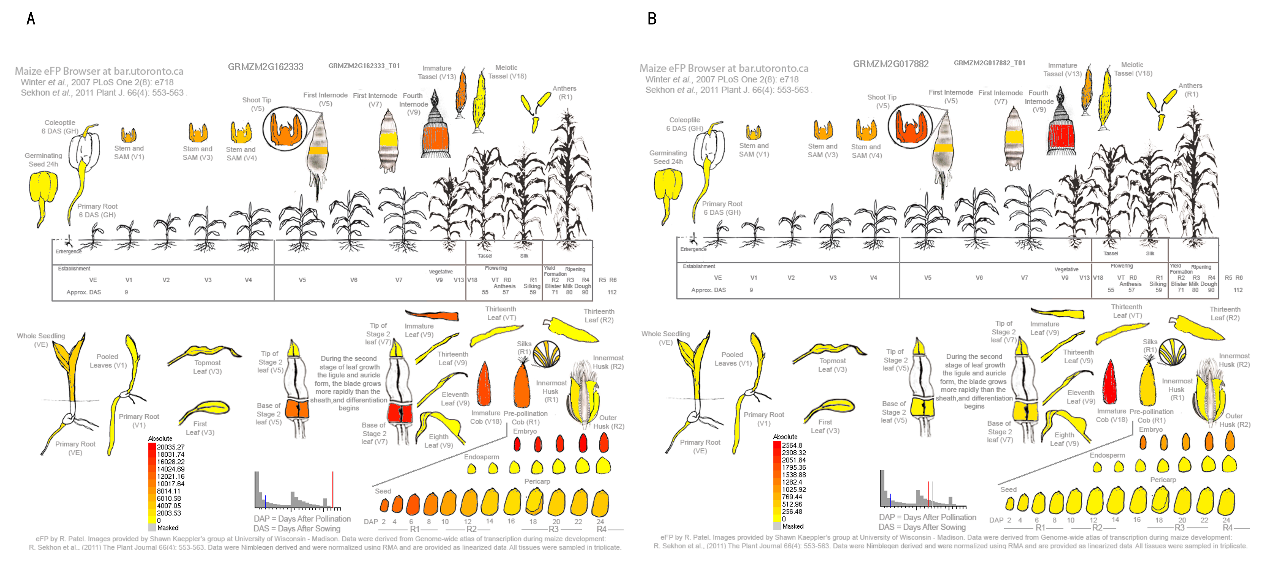

Supplement: Supplementary file 1 [file plants-15-01383-s001.zip › plants-4207105-supplementary/Supplementary File and Figures/Supplementary File/Supplementary Figure.docx]
