# Supplementary figures and images for: Genome-Wide Association Study of Plant and Ear Height in Maize (Zea mays L.) and Identification of Candidate Genes
Source: Plants (Basel). 2026 Apr 30;15(9):1383. doi: 10.3390/plants15091383 (PMC13165044; doi:10.3390/plants15091383)

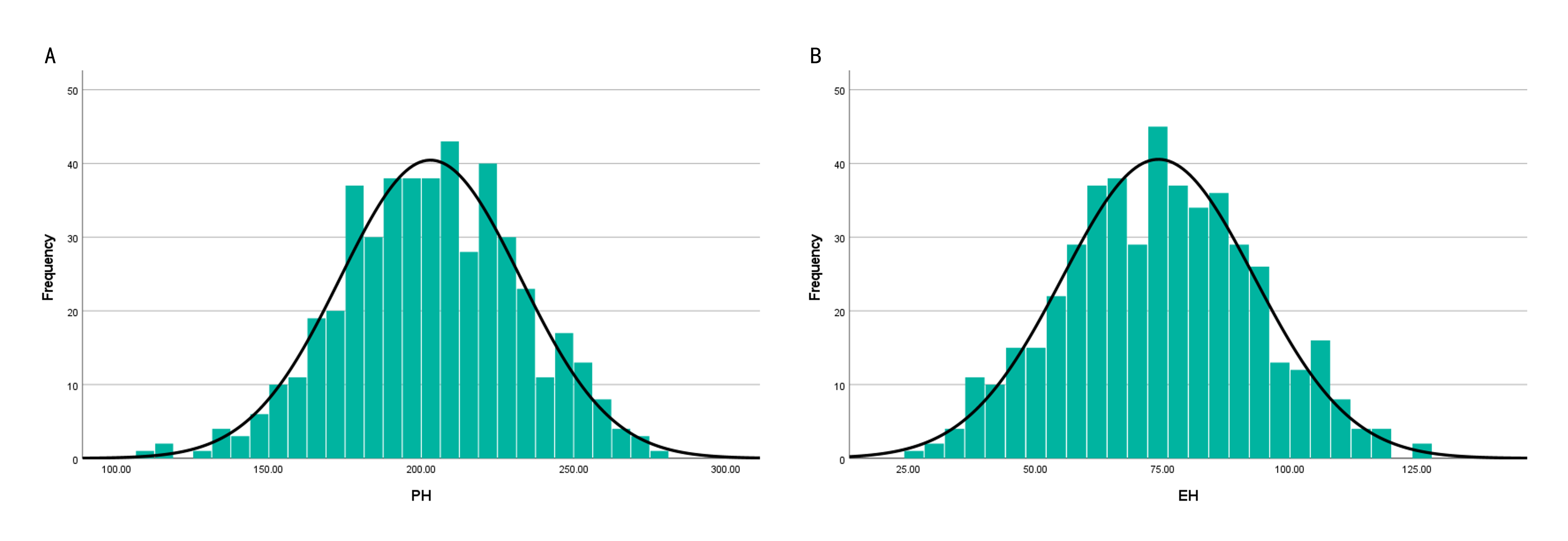

Supplement: Supplementary file 1 [file plants-15-01383-s001.zip › plants-4207105-supplementary/Supplementary File and Figures/Figures/Figure 1.png]

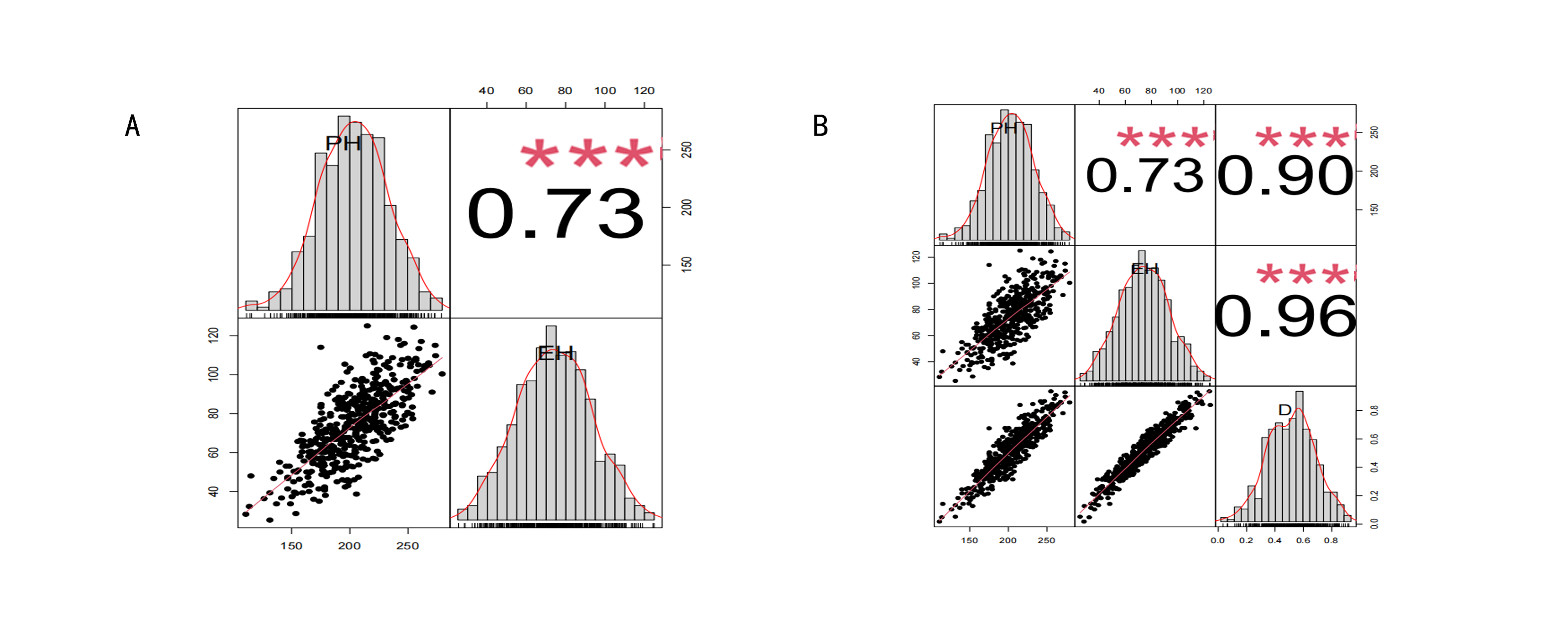

Supplement: Supplementary file 1 [file plants-15-01383-s001.zip › plants-4207105-supplementary/Supplementary File and Figures/Figures/Figure 2.png]

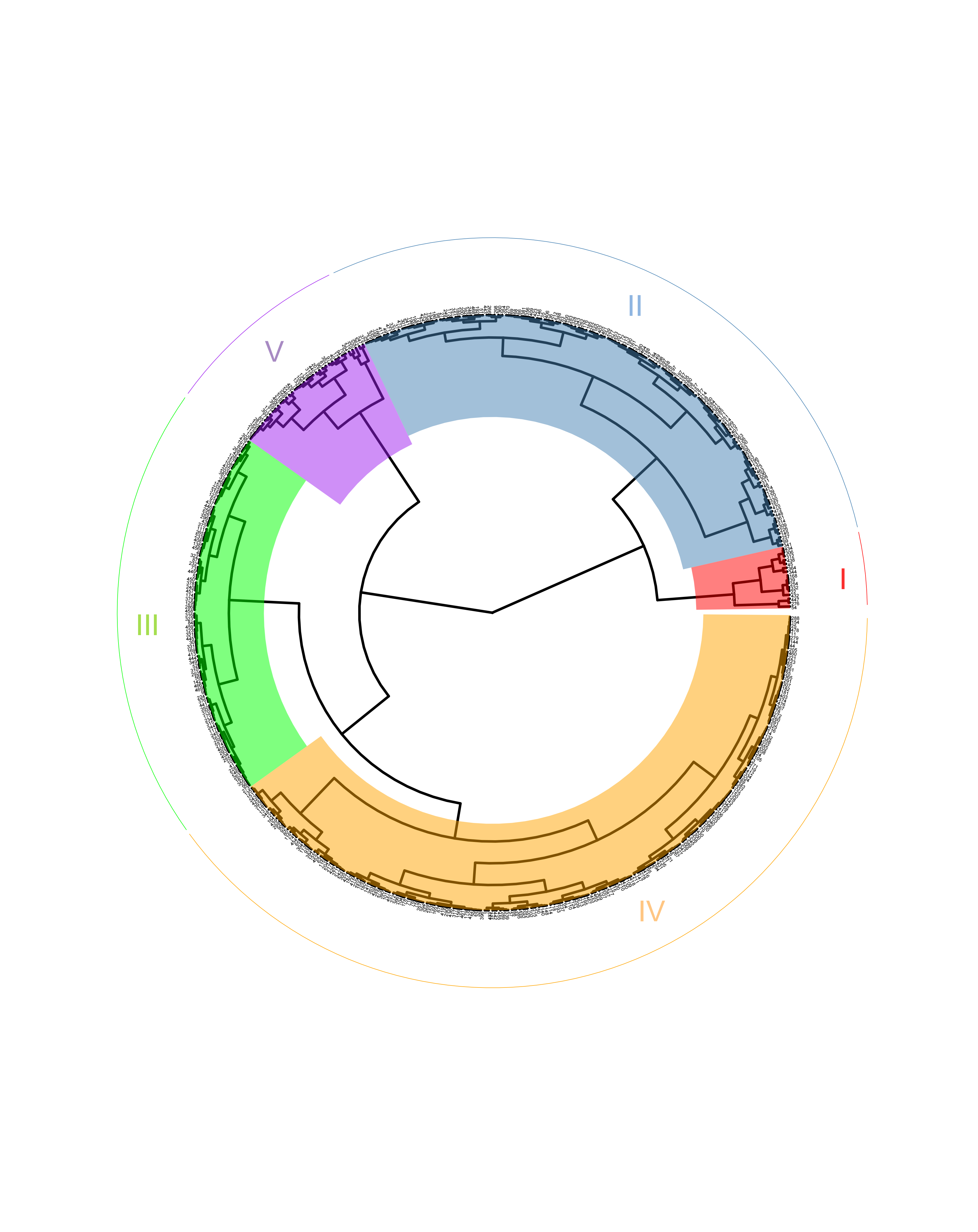

Supplement: Supplementary file 1 [file plants-15-01383-s001.zip › plants-4207105-supplementary/Supplementary File and Figures/Figures/Figure 3.png]

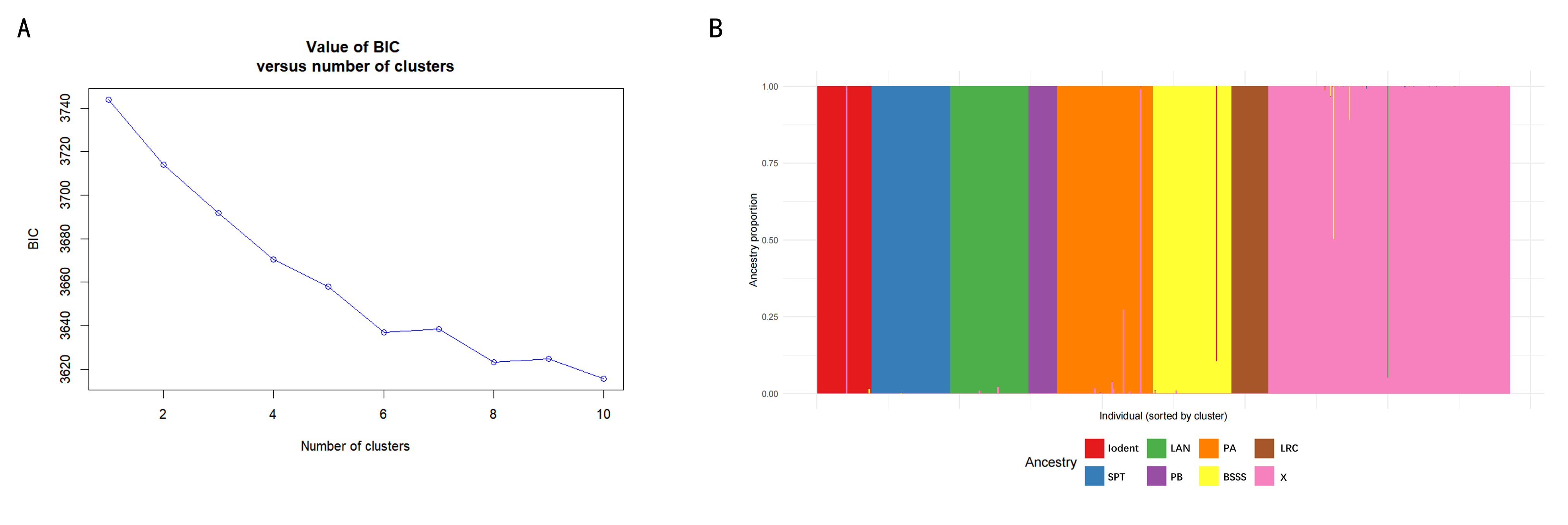

Supplement: Supplementary file 1 [file plants-15-01383-s001.zip › plants-4207105-supplementary/Supplementary File and Figures/Figures/Figure 4.png]

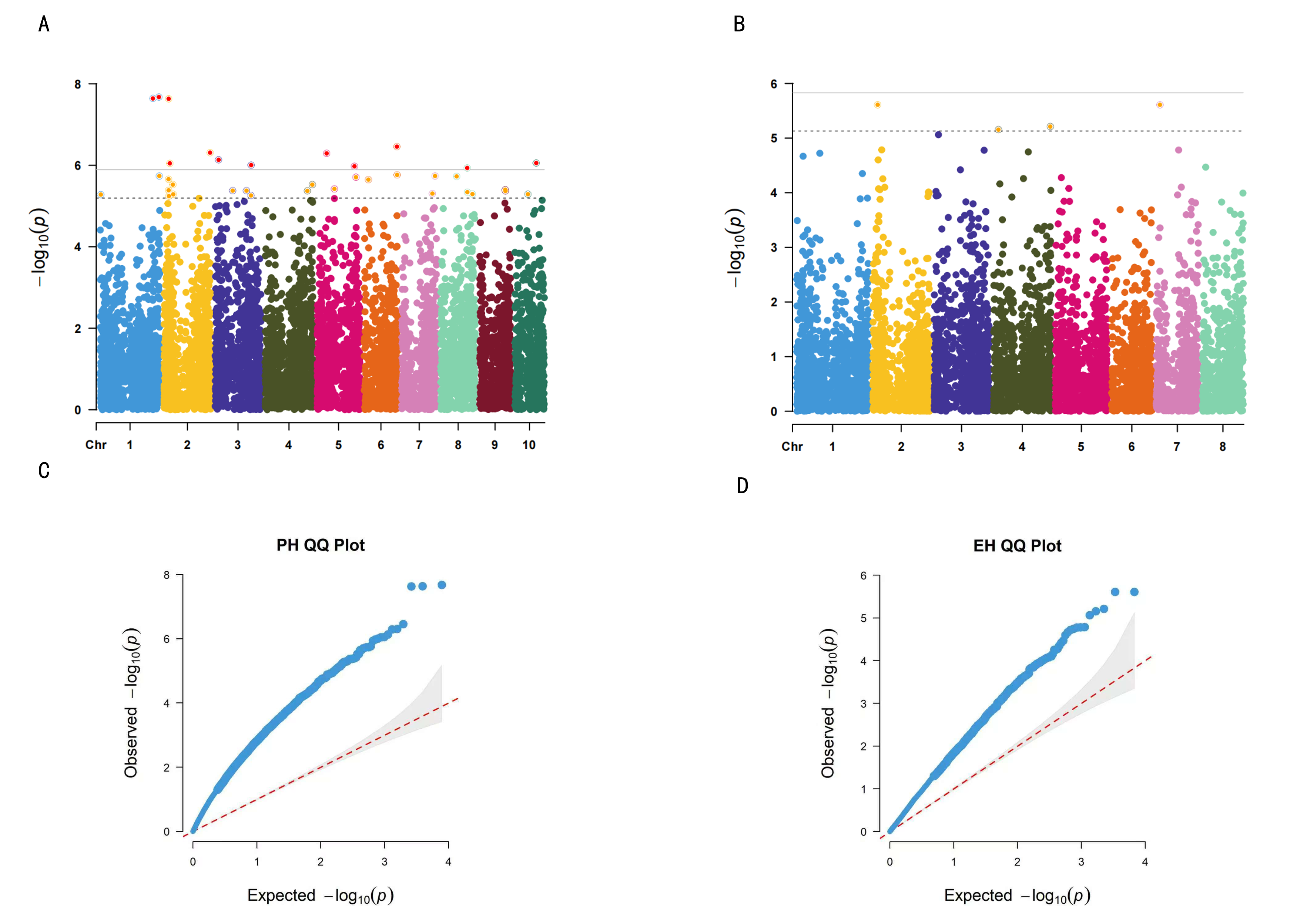

Supplement: Supplementary file 1 [file plants-15-01383-s001.zip › plants-4207105-supplementary/Supplementary File and Figures/Figures/Figure 5.png]

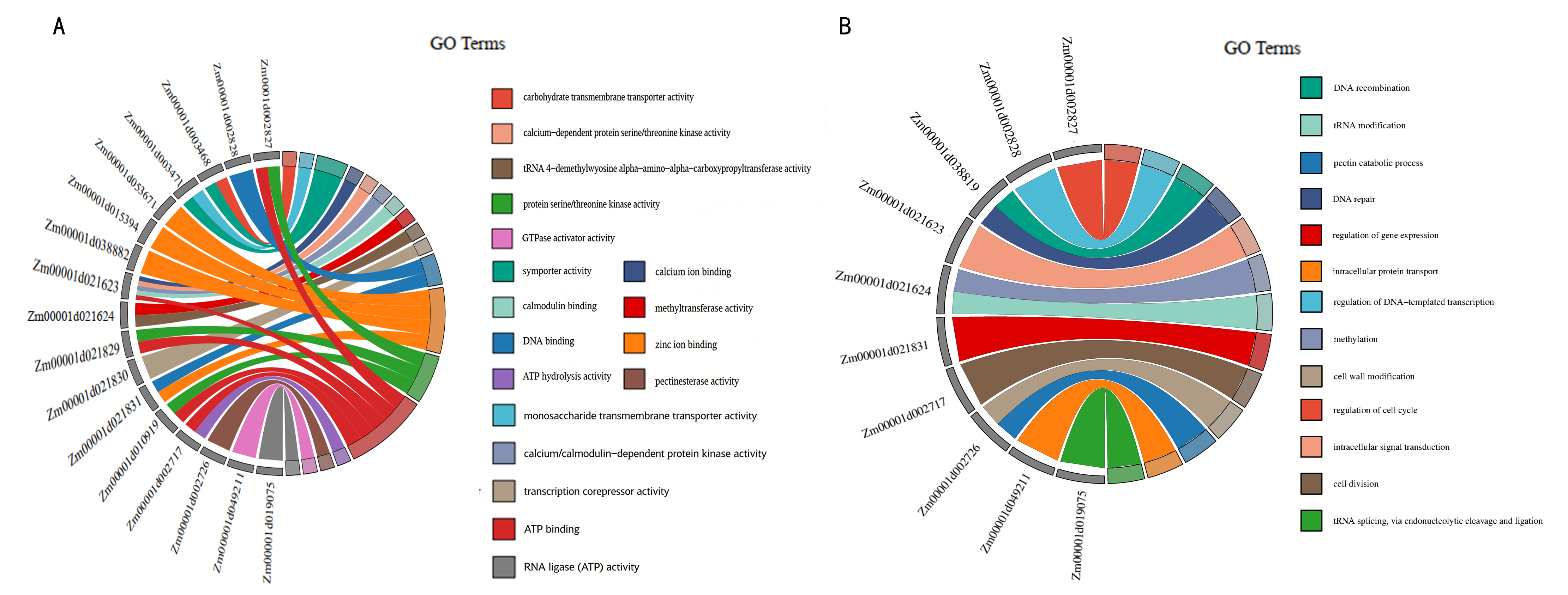

Supplement: Supplementary file 1 [file plants-15-01383-s001.zip › plants-4207105-supplementary/Supplementary File and Figures/Figures/Figure 6.png]

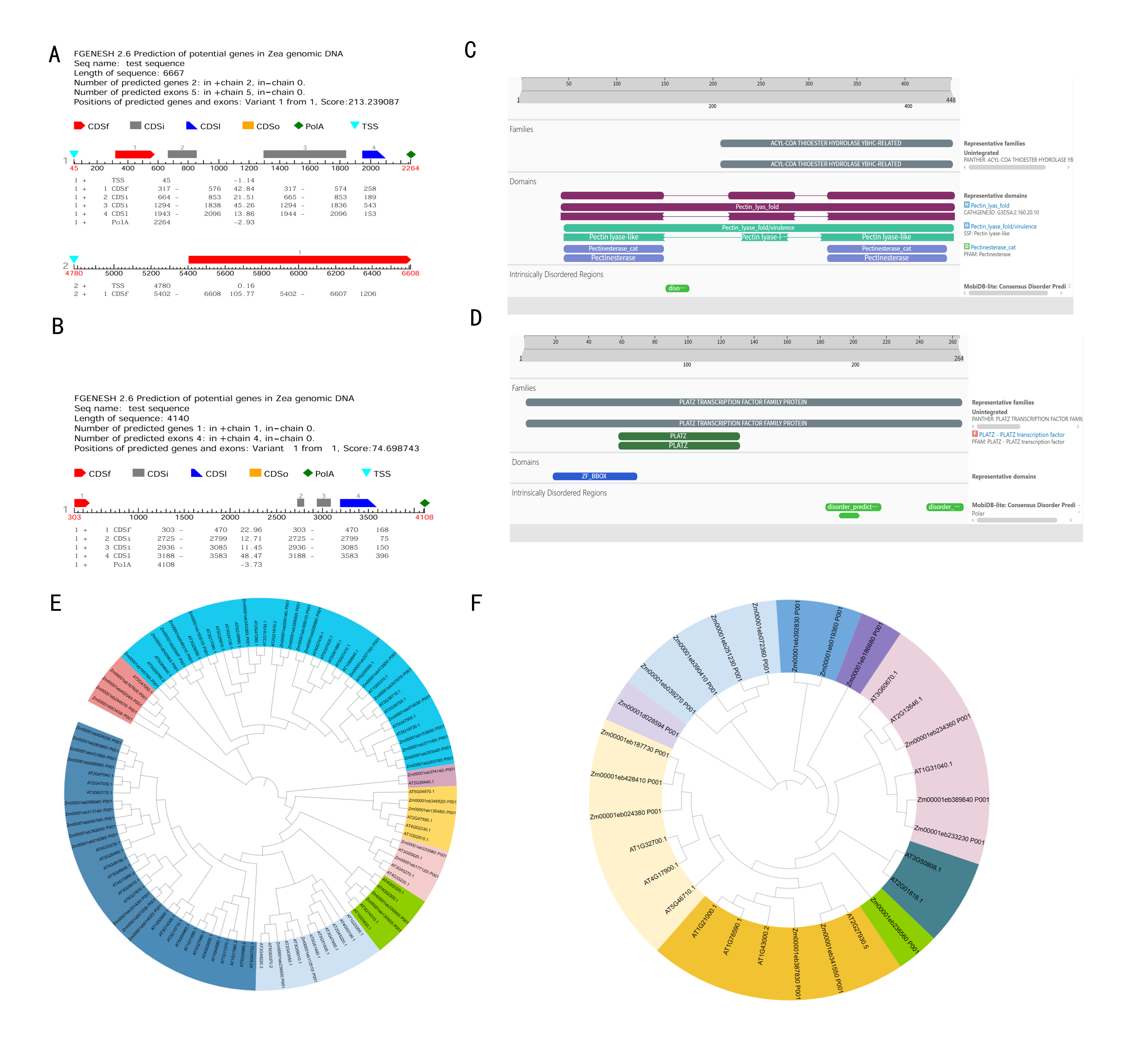

Supplement: Supplementary file 1 [file plants-15-01383-s001.zip › plants-4207105-supplementary/Supplementary File and Figures/Figures/Figure 7.png]

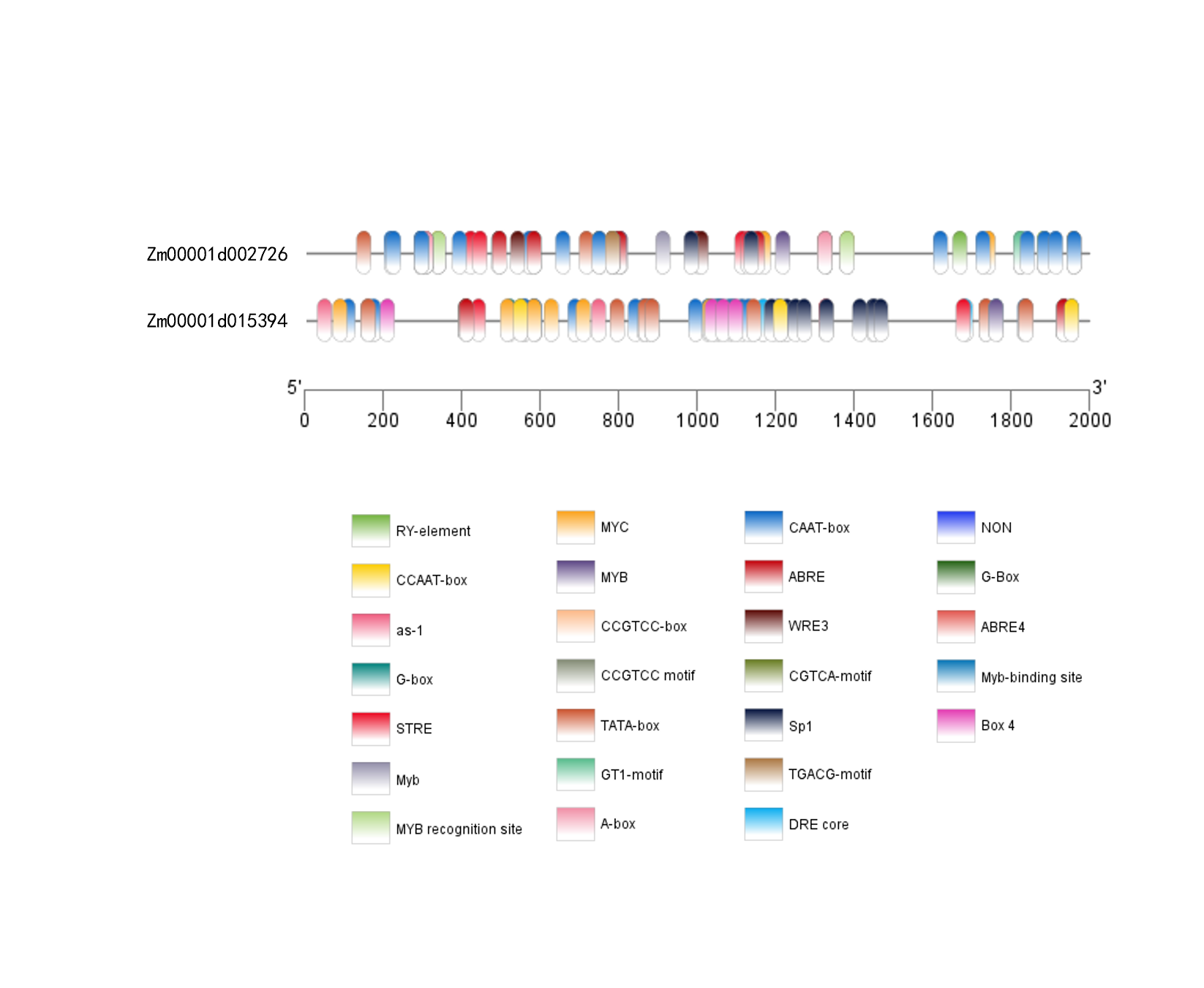

Supplement: Supplementary file 1 [file plants-15-01383-s001.zip › plants-4207105-supplementary/Supplementary File and Figures/Figures/Figure 8.png]
